# Recombinant human erythropoietin ameliorates cognitive dysfunction of APP/PS1 mice by attenuating neuron apoptosis via HSP90β

**DOI:** 10.1038/s41392-022-00998-w

**Published:** 2022-05-06

**Authors:** Hua-Li Wan, Bing-Ge Zhang, Chongyang Chen, Qian Liu, Ting Li, Ye He, Yongmei Xie, Xifei Yang, Jian-Zhi Wang, Gong-Ping Liu

**Affiliations:** 1 Department of Pathophysiology, School of Basic Medicine, Key Laboratory of Ministry of Education of China and Hubei Province for Neurological Disorders, Tongji Medical College, Huazhong University of Science and Technology, Wuhan, China; 2 State Key Laboratory of Biotherapy and Cancer Center, West China Hospital, Sichuan University and Collaborative Innovation Center of Biotherapy, Chengdu, 610041 China; 3 Key Laboratory of Modern Toxicology of Shenzhen, Shenzhen Medical Key Discipline of Health Toxicology (2020-2024), Shenzhen Center for Disease Control and Prevention, Shenzhen, 518055 China; 4 Co-innovation Center of Neurodegeneration, Nantong University, Nantong, J.S. China

**Keywords:** Neurology, Neurological disorders

**Dear Editor**,

Recently, lower hemoglobin/anemia in the elderly is found to be associated with cognitive impairment and Alzheimer’s disease (AD),^1^ implying that erythropoietin (EPO) may be of benefit for AD. As a clinically safe-to-use drug, besides its hematopoietic function, recombinant human EPO (rhEPO) is reported to present multifaceted neuroprotective effects.^2^ Unfortunately, few clinical studies investigated the effect of rhEPO in AD patients. The dose, the therapeutic window, and duration of treatment may be critical influencing factors for clinical applications of EPO.

Abeta deposits appear in the brain began from 6-month-old, accompanied by the decreased EPO and increased EPO receptor (EPOR) levels in the hippocampus of 9-month-old APP/PS1 mice (Supplementary Fig. S1). Next, to illuminate the preventive or therapeutic effects of rhEPO on cognitive deficits, APP/PS1 mice of 3-month-old (preventive experiment) or 6-month-old (therapeutic experiment), were intraperitoneally administered low (2500 IU/kg) or high dose (5000 IU/kg) of rhEPO three times per week until 9-month-old, respectively, followed by cognition measurement (Supplementary Fig. S2a). During the training period of the Morris water maze (MWM), APP/PS1 mice showed the increased latency to find the hidden platform (Supplementary Fig. S2b, f), however, high-dose rhEPO ameliorated the learning ability of APP/PS1 mice at the fourth and fifth day (preventive), or fourth day (therapeutic). Similarly, the memory deficits of APP/PS1 mice were also rescued by preventive treatment of high-dose rhEPO, as shown by less average latency, the increased target platform crossings, and more stay time in the platform quadrant (Supplementary Fig. S2c–e). In the therapeutic experiment, we also found that high-dose rhEPO alleviated the cognitive deficit of APP/PS1 mice as presented by a significant reduction in the escape latency (Supplementary Fig. S2g–i). No significant difference in swimming speed had been detected among mice in all groups, which excluded motor deficits (Supplementary Fig. S3). In a novel object recognition test, high-dose preventive or therapeutic rhEPO significantly increased the preferential or discrimination index to the new object (Supplementary Fig. S2j–m), and attenuated the spine density loss in the neurons of the hippocampal CA3 region of APP/PS1 mice (Supplementary Fig. S4), indicating that rhEPO rescued long-term memory defects before and after the onset of Aβ deposition.

Immunohistochemical and Thioflavin-S staining revealed that either preventive or therapeutic high-dose rhEPO significantly reduced amyloid plaques area fraction, plaque areas, and plaque numbers in the hippocampus and cortex of the transgenic mice (Supplementary Fig. S5a–h). By ELISA, high-dose of rhEPO preventive treatment lowered both soluble and insoluble Aβ40 levels (Supplementary Fig. S5i, j). Similarly, in the therapeutic experiment, 5000 IU/kg rhEPO decreased soluble Aβ40 and Aβ42 levels (Supplementary Fig. S5k, l). There was no positive immunostaining of 6E10 or Thioflavin-S staining in the brain section of WT mice (Supplementary Fig. S6). Moreover, preventive or therapeutic high-dose rhEPO administration predominately reduced β-site APP cleaving enzyme 1 (BACE1) and β-cleavage products (sAPPβ and CTFβ), and high-dose therapeutic rhEPO significantly decreased CTFα level in the cortex (Supplementary Fig. S7).

Neuron loss is a typical pathological marker in AD. We initially detected the dynamic change of neuronal numbers in the brain of 3-, 6-, 9-, and 12-month-old APP/PS1 mice. Neuronal numbers showed an age-dependent decrease with a significant reduction at 9- and 12-month-old in the hippocampal CA3 region, while exhibited a tremendous decrease until 12-month-old in the hippocampal CA1 and DG regions, and cortex (Supplementary Fig. S8). By immunofluorescence, Nissl staining, and Western blotting, (preventive or therapeutic) high-dose rhEPO reversed neuron loss and the increased cleaved-caspase3 level in the hippocampal CA3 area of APP/PS1 mice (Fig. 1a–f and Supplementary Fig. S9). To further evaluate the anti-apoptotic effects of rhEPO, proteomic analysis was performed. 113 differentially expressed (DE) proteins were found, and the heat map showed two reversed expression clusters after rhEPO treatment, which were marked with a blue dashed box (Fig. 1g). The biological process clarified that the cluster of rhEPO-induced downregulated proteins was enriched in metabolic, and oxidation-reduced, processes, etc., and the cluster of up-regulated proteins was focused on protein folding and erythrocyte development, and so on (Supplementary Fig. S10a, b). String analysis showed, interacting DE proteins were involved in the negative regulation of the apoptotic process (Supplementary Fig. S10c). Molecular complex detection (MCODE) revealed that Hsp90aa1 and Hsp90ab1 were closely connected regions in the whole DE protein–protein interaction (PPI) network (Supplementary Fig. S10d). Nineteen DE proteins were found to share in compared groups of rhEPO-treated (APP/PS1-low, -high) vs. the control APP/PS1 mice (APP/PS1-NS) by Venny analysis. Among them, 8 DE proteins demonstrated a dose-effect relationship, including Hsp90aa1 and Hsp90ab1, which are involved in the negative regulation of the apoptotic process (Fig. 1h, i).

**Fig. 1:**
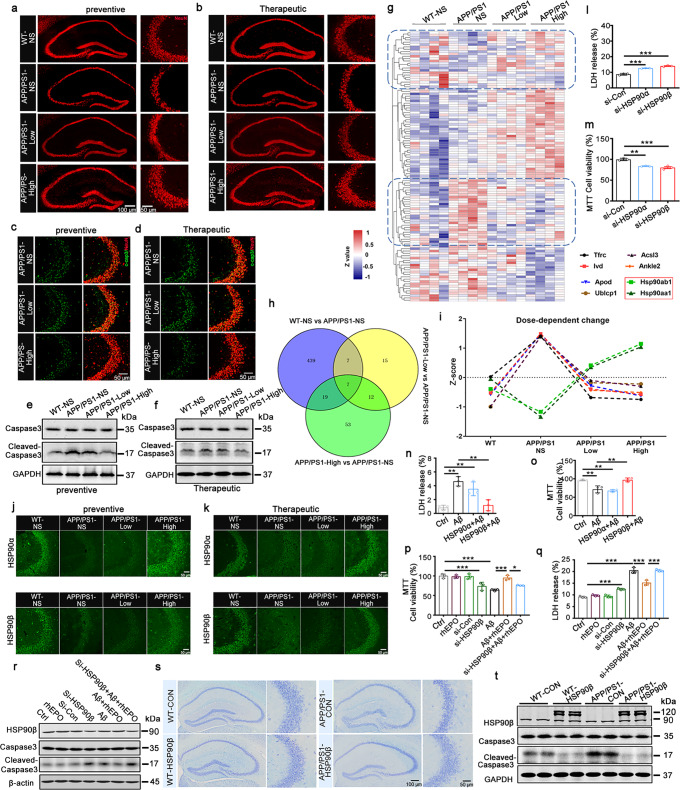
rhEPO attenuated neuronal loss via HSP90β. **a**, **b** Representative images of NeuN immunostaining in the hippocampus of the mice in the prevention (**a**) and treatment experiments (**b**). **c**, **d** Representative images of Neuronal apoptosis detected by immunofluorescence using anti-cleaved-caspase3 (c-cap3) antibody in hippocampal CA3 in the prevention (**c**) and treatment experiments (**d**). **e**, **f** Western blotting for Caspase3 and Cleaved-caspase3 in the hippocampal CA3 in the prevention (**e**) and treatment experiments (**f**). **g** The heat map of total differentially expressed (DE) proteins after rhEPO treatment, red represents high expression abundance, and dark blue represents low expression abundance. **h** Venny analysis to find the shared proteins among different compared groups after rhEPO treatment. **i** The dose-dependent-change of shared proteins among compared groups of rhEPO-treated mice vs untreated mice analyzed by Venny analysis. The proteins abundance was shown as *Z*-score. **j**, **k** Representative images of HSP90α and HSP90β immunostaining in the hippocampal CA3 in the prevention (**j**) and treatment experiments (**k**). **l**, **m** Knockdown of HSP90α or HSP90β in N2a cells promoted cell apoptosis detected by LDH assay (**l**) and MTT (**m**). **n**, **o** Overexpression of HSP90β in N2a cells attenuated cytotoxicity induced by Aβ detected by LDH assay (**n**) and MTT (**o**). **p**–**r** Knockdown of HSP90β attenuated rhEPO-induced cytoprotective effects against Aβ in N2a cells, which were detected by LDH assay (**p**), MTT (**q**), and cleaved-caspase3 was detected by Western blotting (**r**). **s**, **t** AAV-HSP90β-mcherry virus (1.5 × 10^13^v.g/ml) was stereotaxically injected into the hippocampal CA3 of 7.5-month-old C57 and APP/PS1 mice, neuronal number detected by Nissl staining in the hippocampal CA3 region (**s**), caspase3 or cleaved-caspase3 level was detected by Western blotting. Data were presented as mean ± SD. *p < 0.05, **p < 0.01, ***p < 0.001. *N* = 3 per group

As HSP90 was reported to regulate cell proliferation and differentiation, and inhibit apoptosis,^3^ we focused our study on HSP90 protein. HSP90α (Hsp90aa1) or HSP90β (Hsp90ab1) was highly expressed in the CA3 region (Supplementary Fig. S11), and APP/PS1 mice showed a reduced HSP90α or HSP90β level in the hippocampal CA3 region, which reversed by high dose of rhEPO (Fig. 1j, k). And a high dose of rhEPO also reduced p-p38 and p-JNK levels (Supplementary Fig. S12d, e). The reduced HSP90α or HSP90β, and increased cleaved-caspase3 level were also found in N2a cells treatment with Aβ (Supplementary Fig. S12a, b). HSP90α or HSP90β knockdown increased LDH release, decreased cell survival, and enhanced cleaved-caspase3 level (Fig. 1l, m and Supplementary Fig. S12c), with activated JNK/P38 (p-JNK/p-P38 increased) signaling pathway, which similar to in vivo data (APP/PS vs. WT, Supplementary Fig. S12d, e). However, overexpression of HSP90β, but not HSP90α, attenuated two common cell apoptosis inducers staurosporine (STP) or Aβ-induced cell cytotoxicity as presented by decreased LDH, increased cell survival, and reduced cleaved-caspase3 level (Fig. 1n, o, Supplementary Fig. S12f–i). Moreover, HSP90β knockdown reversed rhEPO protective effects against Aβ-induced cytotoxicity, which is shown as the increased LDH release and cleaved-Caspase3 level, and the decreased cell viability (Fig. 1p–r).

To further verify the critical role of HSP90β in mediating the neuroprotective effects of rhEPO in vivo, the AAV-mCherry-HSP90β virus was injected into the hippocampal CA3 region of 7.5-month-old APP/PS1 mice, and the cognitive performance was assessed at 9-month-old (Supplementary Fig. S13a–c). By MWM test, HSP90β overexpression ameliorated learning defects as shown by the decreased escape latency in fifth day, and improved memory abilities as presented by less average latency to reach the target quadrant and more time stayed in the platform quadrant (Supplementary Fig. S13d–h). Using a novel object test, HSP90β overexpression also ameliorated long-term memory defects as demonstrated by more time exploring the new object (Supplementary Fig. S13i). Nissl staining presented that HSP90β overexpression attenuated hippocampal CA3 neuron loss (Fig. 1s, Supplementary Fig. S14a). Overexpressing HSP90β also reduced cleaved-caspase3 level, inactivated the JNK/p38 pathway (Fig. 1t and Supplementary Fig. S14b–e), and attenuated the spine density reduction of neurons in the hippocampal CA3 region of APP/PS1 mice (Supplementary Fig. S14f, g). These data suggested that HSP90β overexpression ameliorates neuronal and synaptic loss.

Finally, high-dose preventive not therapeutic rhEPO treatment attenuated Aβ-induced astrocytosis and increased neovascularization in the hippocampus of APP/PS1 mice (Supplementary Figs. S15, S16). By the way, hemoglobin contents and hematocrit in peripheral blood had no significant change after rhEPO preventive treatment (Supplementary Fig. S17), which was different from other studies.^4^ The reasons for the difference may be related to the dose, duration of administration, and so on, which waiting for further study. And there were no side effects in peripheral organs after high-dose EPO treatment (Supplementary Fig. S18).

In summary, we found that rhEPO treatment attenuated JNK/P38 pathway associated apoptosis via upregulation of HSP90β, reduced Aβ load, and reversed dendritic spine loss, together contributing to ameliorating the cognitive impairment of APP/PS1 mice (Supplementary Fig. S19). Preventive treatment also promoted angiogenesis and inhibited neuroinflammation. Our study revealed that early prevention with high dose rhEPO treatment brought better cognitive improvement, which provides new clues for rhEPO in clinical trials for AD.

## Supplementary information

Supplementary Materials

## Data Availability

All data needed to evaluate the conclusions in the paper are present in the paper or the Supplementary Materials. Materials described in the study are either commercially available or on request from the corresponding author.
