# Supplementary material for: Recombinant human erythropoietin ameliorates cognitive dysfunction of APP/PS1 mice by attenuating neuron apoptosis via HSP90β
Source: Signal Transduct Target Ther. 2022 May 6;7:149. doi: 10.1038/s41392-022-00998-w (PMC9076625; doi:10.1038/s41392-022-00998-w)
Supplement: Supplementary file 1 — Supplementary Materials [file 41392_2022_998_MOESM1_ESM.docx]

**Recombinant human erythropoietin ameliorates cognitive dysfunction of APP/PS1 mice by attenuating neuron apoptosis via HSP90β**

Hua-Li Wan ^a,1^, Bing-Ge Zhang ^a,1^, Chongyang Chen^a,1^, Qian Liu^a^, Ting Li^a^, Ye He^a^, Yongmei Xie^c^, Xifei Yang^d^, Jian-Zhi Wang^a^, and Gong-Ping Liu^a,b*^

^a^Department of Pathophysiology, School of Basic Medicine, Key Laboratory of Ministry of Education of China and Hubei Province for Neurological Disorders, Tongji Medical College, Huazhong University of Science and Technology, Wuhan, China.

^b^Co-innovation Center of Neurodegeneration, Nantong University, Nantong, J.S., China.

^c^Sichuan Univ, West China Hosp, State Key Lab Biotherapy & Canc Ctr, Dept Lab Med, Chengdu 610041, Peoples R China.

^d^Key Laboratory of Modern Toxicology of Shenzhen, Shenzhen Medical Key Discipline of Health Toxicology (2020-2024), Shenzhen Center for Disease Control and Prevention, Shenzhen, 518055, China.

^1^These authors contributed equally to this work.

^*^Corresponding author: Dr. Gong-Ping Liu ([liugp111@mail.hust.edu.cn](mailto:liugp111@mail.hust.edu.cn))

**This PDF files includes:**

Extended Discussion

Materials and Methods

Supplementary Figures. S1 to S19

Supplementary Tables S1

**Extended Discussion**

In the study, HSP90α and HSP90β have the same expression changes during many processes, including treatment of high-dose rhEPO and treatment of N2a cells with Aβ. However, they have different functions on the rhEPO-induced cell survival. Inducible form HSP90α and the constitutive form HSP90β are two main isoforms of HSP90, which share 85% homology and are both found in the cytoplasm^1^. However, they have different function in the regulation of cell survival, as HSP90α involved in growth promotion, cell cycle regulation, stress-induced cytoprotection, and HSP90β functioned in early embryonic development, germ cell maturation, cytoskeletal stabilization, cellular transformation, signal transduction, and long-term cell adaptation^2^. In short, Hsp90α emerges as a fast-response, cytoprotective isoform, while Hsp90β seems to be associated with long-term cellular adaptation and facilitates cellular evolution. These functional differences are likely due to their nucleotide sequence variations in non-coding regions, which allows for precise and specific regulation through interaction with selective transcription factors. Moreover, their subtle changes in amino acid sequences also allow for unique post-translational modifications^3^. In addition, HSP90α tends to dimerize more frequently as compared to HSP90β^2^.

Most researchers and clinicians think that, high dose rhEPO can be tolerated by animal, but not by all individuals, especially those with co-morbid diseases such as hypertension, neoplasms and congestive heart failure. As the neuroprotective effect of rhEPO probably separates from its hematopoietic activity, developing non-erythropoietic derivatives, analogs, or mimetic peptides of EPO with neuroprotective effects, may be potential choices for clinical application. Intranasal administration also attracts significant interests^4^.

**Materials and Methods**

**Antibodies and reagents**

The used antibodies were listed in the Supplementary Table S1. The mCherry-HSP90α plasmid, encoding human HSP90α, and mCherry-HSP90β plasmid, encoding human HSP90β were come from Addgene. SiHSP90α (sc-35607) and siHSP90β (sc-35609) were purchased from Santa Cruz, β-Amyloid (1-42) human was come from China peptides (Shanghai), staurosporine (STP, HY-15141) was from MedChemExpress. Human Aβ1-40 (E-EL-H0542c) and Aβ1-42 (E-EL-H0543c) ELISA Kit were purchased from Elabscience (Wuhan, China).

**Animals**

Female APP/PS1 transgenic mice were come from Model Animal Research Center of Nanjing University. All animals were bred in the Animal Center of Tongji Medical College, Huazhong University of Science and Technology. Transgenic mice and wild-type littermate mice were randomly allocated and kept at 24 ± 2 ºC on daily 12/12 h light and dark cycles with accessible food and water. 3-month-old mice were used for the preventive experiment, and 6-month-old mice were used for the therapeutic study. The mice were treated with recombinant human EPO (2,500 IU/kg or 5,000 IU/kg; three times/week; 3SBIOINC, China) in normal saline (NS) or an equal volume of NS alone by intraperitoneal injection. All behavioral, histologic and biochemical evaluations were performed until 9 months old.

**Stereotaxic brain injection**

AAV-mCherry-HSP90β (1.5×10^13^ v.g./ml) or the control AAV-mCherry (1.2×10^13^ v.g./ml), which were driven by SYN-promoter, were come from OBiO Biologic Technology Co., Ltd. For brain stereotactic injection, 7.5-month-old mice were bilaterally injected with AAV-mCherry-HSP90β or its control virus into the hippocampal CA3 area (AP ±2.0, ML -1.5, DV -2.0) with at a rate of 0.10 μl/min, after the animals were positioned in a stereotaxic instrument. The needle syringe was left in place for 15 min before being withdrawn. The injection had no significant effects in the normal activity or did not increase the death rate of the animals.

**Morris water maze (MWM) test**

MWM test was carried out as described previously^5^. The water maze was divided into four quadrants and filled with water with a platform placed in one quadrants. For spatial learning, mice were trained to find the hidden platform for 5 consecutive days, 3 trials per day with a 30-s interval from 10:00 am to 17:00 pm. On each training trial, the mice started from one quadrants with facing the wall of the pool and ended when the animal climbed on the platform. The mice were guided onto the platform and stayed for another 30 s, if the mice did not find the platform within 60 s. The spatial memory tested on one day (day 7) after training and the platform was removed. A video camera, which fixed to the ceiling and 1.5 m from the water surface, was used to record the swimming path, the time used to find the platform (latency) and pass through the previous platform quadrant, etc.

**Novel object recognition test**

The mice were granted 5 min to habituate the arena (50 cm × 50 cm container) without objects one day prior to the test. Two same objects A and B were placed on opposite sides of the test area, and then, the mice reentered into the arenas and explored for 5 min. After one hour, a new object C, which had the same material and size but different in shape, was replaced with one of the two objects, and the mice were explore the objects for 5 min. Between each habituation period, 70% ethanol was used to clean, and dry paper was used to wipe the arena and objects. In order to allow ethanol evaporation, at least 5 min were left. During the test, a video camera was positioned above the arena and recorded the behavior. The preferential index was calculated by TC/ (TA + TC). The discrimination index was calculated by (TC-TA)/(TA + TC)^6^.

**Western blotting**

Briefly, brain tissues were lysed with RIPA buffer at 4 ºC for 10 min, and then, the extract was mixed with sample buffer (3:1, v/v) and boiled for 10 min, followed by centrifuged at 12,000 × g for another 10 min. The supernatant was collected and used for Western blotting. Equal amounts of protein extracts were separated by 10% sodium dodecyl sulfate polyacrylamide gel electrophoresis, and then transferred to nitrocellulose membranes. After blocked with 5% BSA, the membranes were incubated with primary antibodies (Supplementary Table S1) at 4 ºC overnight. And then, an Odyssey secondary antibody (1:10,000; Odyssey) was added to the membranes and incubated for 1 h at 25 ºC. Finally, an Odyssey Infrared Imaging System (LI-COR Biosciences, Lincoln, NE, USA) was used to visualize the immunoreactive bands.

**Immunofluorescence**

Fixed brain slices were permeabilized with PBS containing 0.5% Triton X-100 for 30 min at room temperature, followed by incubated in blocking buffer (0.1% Triton X-100, 5% BSA in PBS) for another 30 min at 37 °C. After incubated with primary antibodies at 4 °C for 24 h, the slices were added with the secondary antibodies and incubated for 1 h at 37 °C. Finally, DAPI was added to the slices for 10 min at room temperature in the dark. Images were acquired using a laser confocal microscope (710; Zeiss, Germany).

**Nissl staining**

After washed with PBS for 3 times, the frozen sections were put on the slides and dried naturally, followed by submerged in 0.5% cresyl violet solution for ~ 5 min at 25 °C. The slices were color separation with 70% alcohol, and then dehydrated with 80% and 95% alcohol for 2 min, respectively. Next, the slices were dehydrated again with absolute alcohol (5 min × 2), and cleared in xylene for 10 min × 2. A microscope (Nikon, 90i, Tokyo, Japan) was used to observe the images.

**Proteomics**

The procedure of proteomic was referenced from previous published papers^7-9^. In briefly, the hippocampus was lysed with 8M urea buffer (1× PBS, pH = 8.0) and the protein concentration was determined by the BCA protein assay reagent kit. A total 50 µg protein per sample was incubated with DTT (10 mM dithiothreitol) for 1 h at 55 °C, followed by incubation with IAA (25 mM iodoacetamide) for 1 h at 37 °C in the dark, and then digested with trypsin for 14 h. After digestion, the peptide was desalted and labeled with TMT tags (Thermo Fisher, NJ, USA), and then separated by high performance liquid chromatography (HPLC) to collect final 15 fractions, followed by performed with liquid chromatography (LC)-mass spectrometry (MS)/MS analysis. Then, the UniProt-Mus musculus database was used for protein search by Proteome Discoverer 2.1 software. The Perseus software was used to calculate the p-value and the differential expressed (DE) protein was set to p < 0.05 among two compared groups. The Database for Annotation, Visualization and Integrated Discovery (DAVID), KEGG database and RStudio were used for bioinformatics analysis.

**Golgi Staining**

Golgi staining was performed by using a FD Rapid Golgi Stain Kit (FD neurotechnology, PK401). Briefly, the mice were sacrificed and then perfused transcardially with PBS for 5 min, followed with PBS containing 4% PFA for another 15 min. After dissection, the brain tissues were immersed in the impregnation solution at 25 °C, and the impregnation solution was replaced after 4 h. After two weeks, Solution C was incubated with the samples for 2 days at 4 °C, and replaced after 4 h. All procedures were performed in the dark. After the samples were sectioned and sections were mounted with Solution C, the slices were rinsed in distilled water and then, placed in a mixture of Solution D:E:distilled water for 10 min. The slices were immersed in gradient alcohol, followed by cleared in xylene (Sinopharm Chemical Reagent, 10023418) for 5 min × 3 and cover-slipped with Permount solution. The images were obtained using Olympus BX60 (Tokyo).

**Immunohistochemistry**

The brain slices were washed with PBS containing 0.2% Tween 20 (PBST) for 3 × 10 min. And then the slices were incubated with 3% H_2_O_2_ in absolute ethanol for 10 min, followed by 0.5% Triton X-100 for 30 min. Blocking buffer (5% BSA), which block nonspecific sites, was used to incubate with the slices for 30 min at room temperature. After incubation with primary antibodies at 4 °C for 24 h, the slices were incubated with the secondary antibodies at 37 °C for 1 h. HistostainTM-SP kits was used to develop the immunoreaction and the immunoreaction was visualized with diaminobenzidine (brown color). Slices were then dehydrated through a graded ethanol series, mounted on glass slides, and sealed with glass coverslips. The images was observed with a microscope (Olympus BX60, Tokyo, Japan).

**Thioflavin-S staining**

The brain slices were washed with PBS, and continued to wash by graded alcohol (50, 70 and 80%) for 1 min each. Sections were stained with 0.1% thioflavin-S (Sigma) in 80% ethanol for 10 min in the dark, followed by three washes in 70% ethanol, and one wash with PBS^10^. Images at different subregions were captured by a laser confocal microscope (710; Zeiss, Germany).

**HE staining**

The paraffin sections first were put in xylene for dewax and were moved into different concentrations of alcohol for rehydration. After rinsed with running water for 2 min, slices were moved into hematoxylin for 5 min (depending on the staining situation, the staining time can be increased or decreased appropriately), then rinsed with running water again. The slices were removed into the differentiation fluid (1% hydrochloric acid alcohol) for a few seconds and rinsed with running water, then were removed into the eosin for 1 min (depending on the staining situation) and rinsed with running water. The slices were immersed in gradient alcohol, followed by cleared in xylene and cover-slipped with neutral resins. The images were obtained using Olympus BX60 (Tokyo).

**Fluorescein isothiocyanate (FITC) perfusion**

Mice first rinsed intravascular blood quickly with precooled PBS (including 10 mM glucose) of pH 7.0, followed by PBS (pH = 7.0) containing 0.1mg/ml FITC and 10 mM glucose for 5 min, then perfused with 4% paraformaldehyde (pH = 8.0) to form thiourea bond between FITC and amino acid, and finally perfused with PBS (pH = 7.0) containing 5U/ml heparin and 10 mM glucose for 2 min. The brains were removed and kept in 4% paraformaldehyde (pH = 8.0) at 4 °C for 24 h, then dehydrated in 30% sucrose (pH = 8.0). After completely sunk, the brains were cut into slice (30 μm thick) with a frozen slicer and preserved in antifreeze solution (PBS: glycerol: ethylene glycol = 5:3:2) at 4 °C ^11^. The images were observed by a laser confocal microscope (710; Zeiss, Germany).

**ELISA**

Brain tissues Aβ was homogenized in a 5 M guanidine HCl, 50 mM Tris-HCl (pH = 8.0) buffer, followed by extracted with 70% (vol/vol) formic acid (FA) solutions^12^. The concentration of Aβ40 or Aβ42 was measured by Human Aβ1-40 (E-EL-H0542c) and Aβ1-42 (E-EL-H0543c) ELISA Kit according to the manufacturer’s instructions (Elabscience, China).

**Thiazole Blue Colorimetry (MTT) assay**

Generally, the human Aβ42 was dissolved in dimethyl sulfoxide, ultrasonically treated, and diluted with medium. Then, the solution was incubated at 4 °C for 24 h and centrifuged at 16,000 g for 20 min to collect the supernatant as oligomerization Aβ^13-15^. MTT cell proliferation and cytotoxicity Assay Kit (Beyotime, China) was used to perform the proliferation assay. Briefly, N2a cells (1 × 10^5^ cells/mL) were plated in 96-well plates and grew overnight. The cells were randomly divided into different groups. After transfected with the plasmids and/or treatment with drugs according to the experimental design, the cells were incubated with 20 µl MTT (5 mg/ml in PBS) at 37 °C for 4 h. MTT-formazan product was dissolved by DMSO, and then measured the absorbance at 490 nm.

**Lactate dehydrogenase (LDH) assay**

Cytotoxicity was evaluated by the quantification of lactate dehydrogenase (LDH) in the culture medium, which was released due to plasma membrane damage. Followed the manufacturer's instructions, LDH level was detected by LDH cytotoxicity assay kit (Beyotime, China)

**Statistical analysis**

All data were collected and analyzed in a blinded manner. Data were expressed as mean ± SD or mean ± SEM. Student’s t-test was used for the comparison between two groups, and the one-way ANOVA or two-way repeated measures ANOVA followed by Bonferroni’s post-hoc test was used to analysis the data among multi-group, with SPSS 12.0 statistical software (SPSS Inc. Chicago, IL, USA). The level of significance was set at *p* < 0.05.

**References**

1. Johnson, J. L. Evolution and function of diverse Hsp90 homologs and cochaperone proteins. *Biochim Biophys Acta*. **1823**, 607-613, (2012).
2. Sreedhar, A. S., Kalmar, E., Csermely, P. & Shen, Y. F. Hsp90 isoforms: functions, expression and clinical importance. *FEBS Lett*. **562**, 11-15, (2004).
3. Zuehlke, A. D., Beebe, K., Neckers, L. & Prince, T. Regulation and function of the human HSP90AA1 gene. *Gene*. **570**, 8-16, (2015).
4. Maiese, K., Chong, Z. Z., Li, F. & Shang, Y. C. Erythropoietin: elucidating new cellular targets that broaden therapeutic strategies. *Prog Neurobiol*. **85**, 194-213, (2008).
5. Morris, R. G., Garrud, P., Rawlins, J. N. & O'Keefe, J. Place navigation impaired in rats with hippocampal lesions. *Nature*. **297**, 681-683, (1982).
6. Chen, C. *et al.* Melatonin ameliorates cognitive deficits through improving mitophagy in a mouse model of Alzheimer's disease. *J Pineal Res*. **71**, e12774, (2021).
7. Chen, C. *et al.* Low-dose oral copper treatment changes the hippocampal phosphoproteomic profile and perturbs mitochondrial function in a mouse model of Alzheimer's disease. *Free Radic Biol Med*. **135**, 144-156, (2019).
8. Gokce, E., Andrews, G. L., Dean, R. A. & Muddiman, D. C. Increasing proteome coverage with offline RP HPLC coupled to online RP nanoLC-MS. *J Chromatogr B Analyt Technol Biomed Life Sci*. **879**, 610-614, (2011).
9. Xu, B. *et al.* Proteomic Profiling of Brain and Testis Reveals the Diverse Changes in Ribosomal Proteins in fmr1 Knockout Mice. *Neuroscience*. **371**, 469-483, (2018).
10. Maiti, P. *et al.* A comparative study of dietary curcumin, nanocurcumin, and other classical amyloid-binding dyes for labeling and imaging of amyloid plaques in brain tissue of 5x-familial Alzheimer's disease mice. *Histochem Cell Biol*. **146**, 609-625, (2016).
11. Miyata, S. & Morita, S. A new method for visualization of endothelial cells and extravascular leakage in adult mouse brain using fluorescein isothiocyanate. *J Neurosci Methods*. **202**, 9-16, (2011).
12. Jiao, S. S. *et al.* Edaravone alleviates Alzheimer's disease-type pathologies and cognitive deficits. *Proc Natl Acad Sci U S A*. **112**, 5225-5230, (2015).
13. Feng, B. *et al.* Planar cell polarity signaling components are a direct target of beta-amyloid-associated degeneration of glutamatergic synapses. *Sci Adv*. **7**, (2021).
14. Yanagisawa, D. *et al.* Keto form of curcumin derivatives strongly binds to Abeta oligomers but not fibrils. *Biomaterials*. **270**, 120686, (2021).
15. Castillo, C. *et al.* Neuroprotective effects of EpoL against oxidative stress induced by soluble oligomers of Abeta peptide. *Redox Biol*. **24**, 101187, (2019).


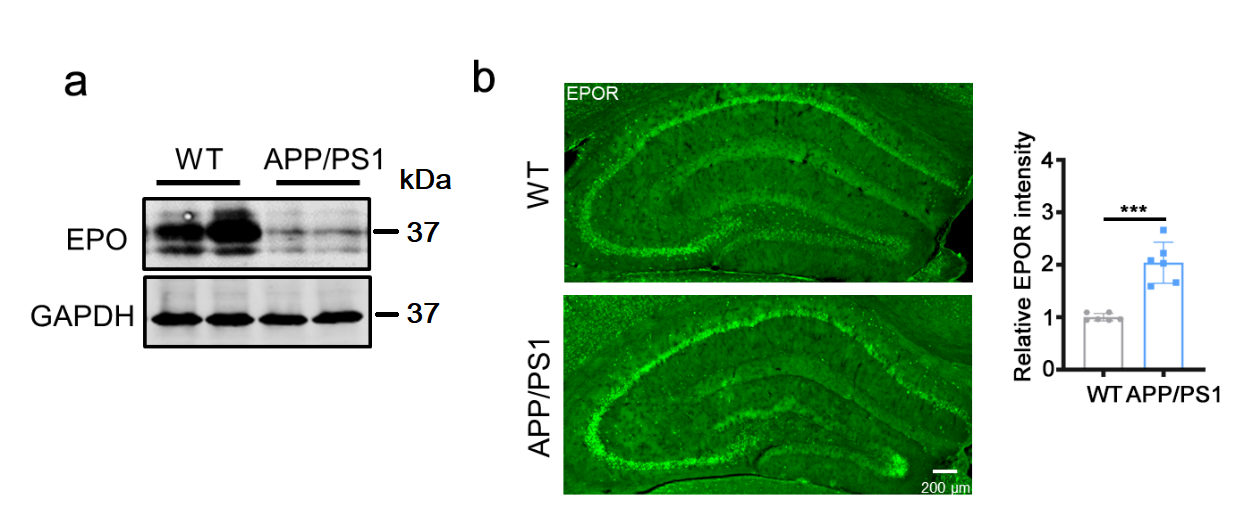
**Supplementary Figures**

**Supplementary Figure S1. EPO level decreased and EPO receptor increased in the hippocampus of APP/PS1 mice**

(a) EPO protein level was decreased in the hippocampus of 9-month-old APP/PS1 mice detected by Western blotting.

(b) EPO receptor (EPOR) level was increased in the hippocampus of 9-month-old APP/PS1 mice analyzed by immunofluorescence.

**
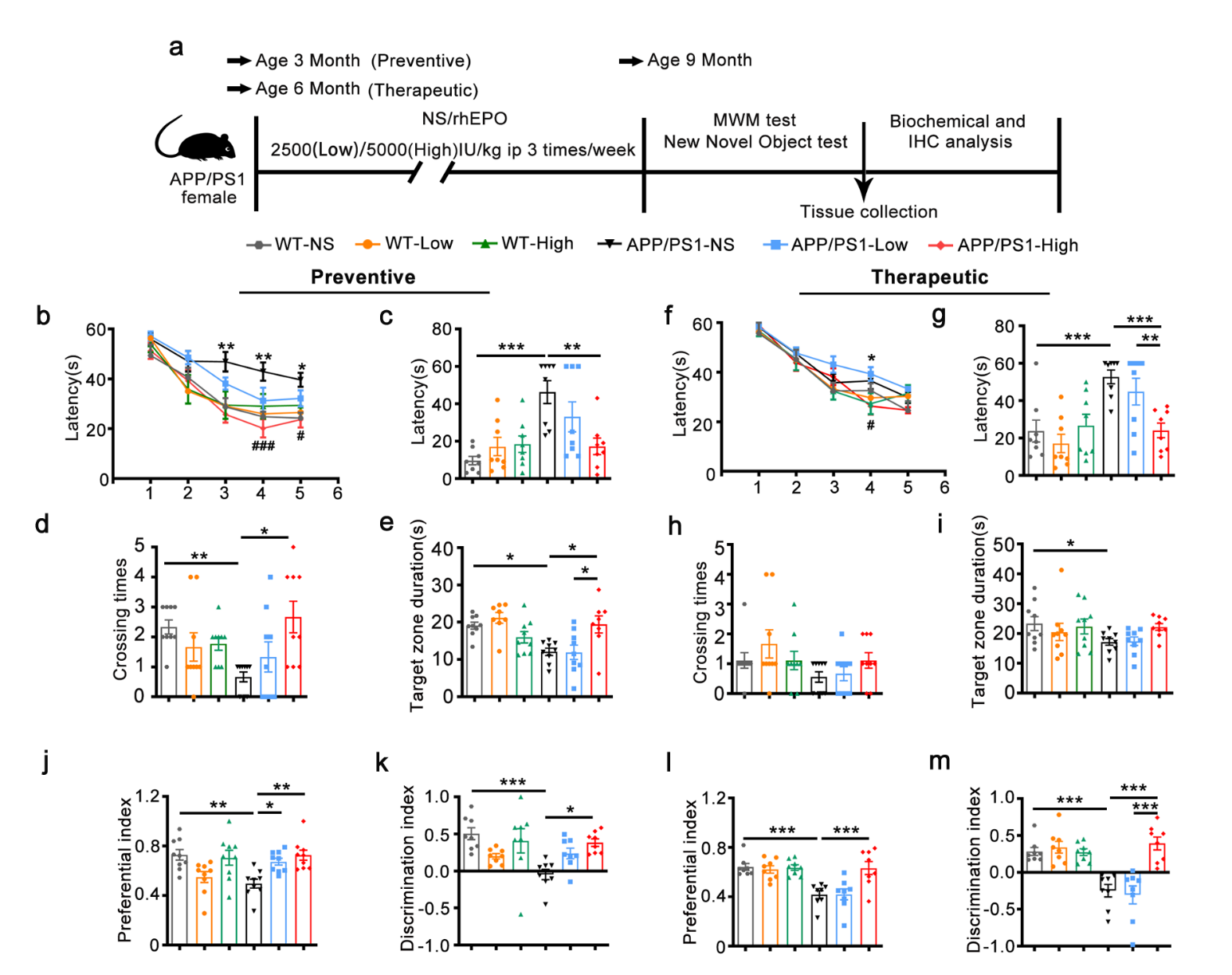
Supplementary Figure S2. rhEPO improved cognitive deficits before and after the onset of Aβ deposition.**

1. Experimental schedules for rhEPO treatment and assessment.

(b-e) Behavior detected by Morris water maze (MWM) test in the prevention experiment.

(b) rhEPO ameliorated spatial learning deficits of APP/PS1 mice shown by the decreased escape latency during 5 consecutive days training. *, *p*<0.05, **, *p*<0.01 *vs* WT-NS (normal saline); #, *p*<0.05, ###, *p*<0.001 *vs* APP/PS1-NS. N = 9 per group.

(c-e) rhEPO treatment ameliorated spatial memory deficits of APP/PS1 mice shown by the decreased latency to reach the platform quadrant (c), crossing times in the platform site (d), and increased time spent in the target quadrant (e) measured at day 7 by removed the platform in MWM test. N = 9 per group.

(f-i) Behavior detected by Morris water maze (MWM) test in the therapeutic experiment.

(f) rhEPO ameliorated spatial learning deficits of APP/PS1 mice shown by the decreased escape latency during 5 consecutive days training in MWM test. *, *p*<0.05 *vs* WT-NS; #, *p*<0.05 *vs* APP/PS1-NS. N = 8 per group.

(g-i) rhEPO treatment ameliorated spatial memory deficits of APP/PS1 mice shown by the decreased latency to reach the platform site (g) measured at day 7 by removed the platform in MWM test; rhEPO treatment had no effect in the crossing time in the platform site (h), and the time spent in the target quadrant (i). N = 8 per group.

(j, k) rhEPO ameliorated cognition impairment of APP/PS1 mice in the prevention experiment shown by increased time spending in exploring the new novel object (preferential or discrimination index) measured at 24 h by Novel object recognition test. N = 8 per group.

(l, m) rhEPO ameliorated cognition impairment of APP/PS1 mice in the therapeutic experiment shown by increased time spending in exploring the new novel object (preferential or discrimination index) measured at 24 h using Novel object recognition test. N = 8 per group.

Data were presented as mean ±SEM. *, *p*<0.05, **, *p*<0.01, ***, *p*<0.001.

**
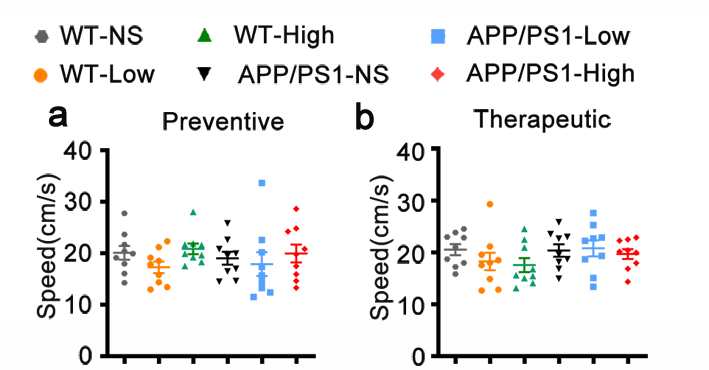
**

**Supplementary Figure S3. rhEPO administration had no effect in the swimming velocity.**

(a) rhEPO had no effect in the swimming speed of the mice in the prevention experiment. N = 9 per group.

(b) rhEPO had no effect in the swimming speed of the mice in the therapeutic experiment. N = 8 per group.

Data were presented as mean ±SEM.

**
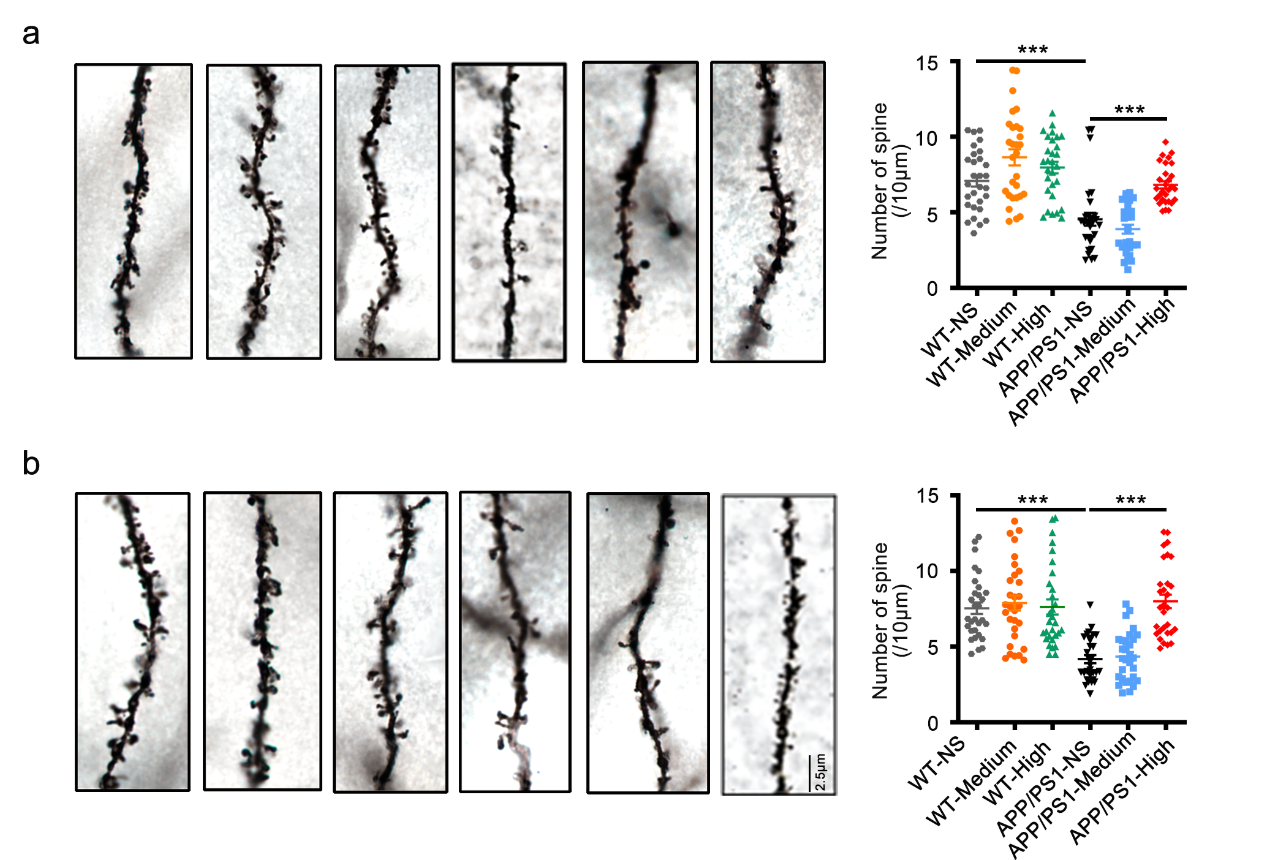
**

**Supplementary Figure S4. rhEPO ameliorated the reduced synaptic density in APP/PS1 mice.**

(a) Preventive treatment with rhEPO reversed the decreased density of dendritic spine detected by Golgi staining. (n=30 neurons from 3 mice for each group).

(b) Therapeutic treatment with rhEPO reversed the decreased density of dendritic spine detected by Golgi staining. (n=30 neurons from 3 mice for each group).

Data were presented as mean ±SD. ***, *p*<0.001.

**
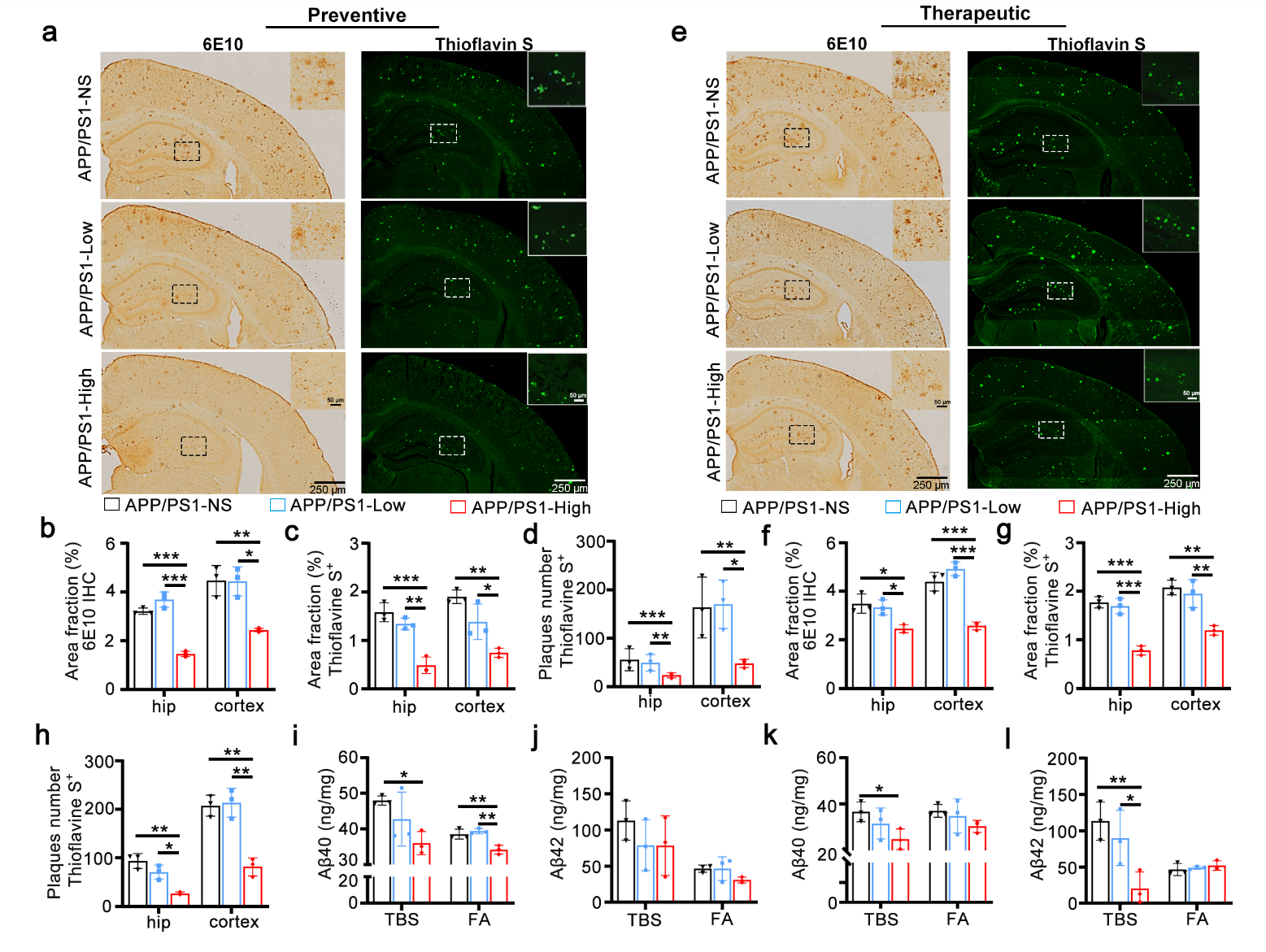
Supplementary Figure S5. rhEPO reduced Aβ burden of APP/PS1 mice.**

(a) 6E10 immunohistochemical and Thioflavin S staining in the brain slices of the mice in the prevention experiment.

(b, c) The quantitative analysis for the area fraction of 6E10-positive plaques (b) and Thioflavin S-positive plaques (c) in the cortex and hippocampus (hip) of the mice in the prevention experiment.

(d) Preventive rhEPO administration reduced Thioflavin S positive plaques number in APP/PS1 mice.

(e) 6E10 immunohistochemical staining and Thioflavin S staining in the brain slices of the mice in the therapeutic experiments.

(f, g) The quantitative analysis for the area fraction of 6E10-positive plaques (f) and Thioflavin S-positive plaques (g) in the cortex and hippocampus (hip) of the mice in the therapeutic experiment.

(h) Therapeutic rhEPO administration reduced Thioflavin S positive plaques number in APP/PS1 mice.

(i, j) Aβ40 or Aβ42 level in TBS and formic acid (FA) fraction of brain homogenates of the mice was detected by ELISA in the prevention experiment.

(k, l) Aβ40 or Aβ42 level in TBS and formic acid (FA) fraction of brain homogenates was detected by ELISA in the therapeutic experiment.

Data were presented as mean ±SD. *, *p*<0.05, **, *p*<0.01, ***, *p*<0.001. N = 3 per group.

**
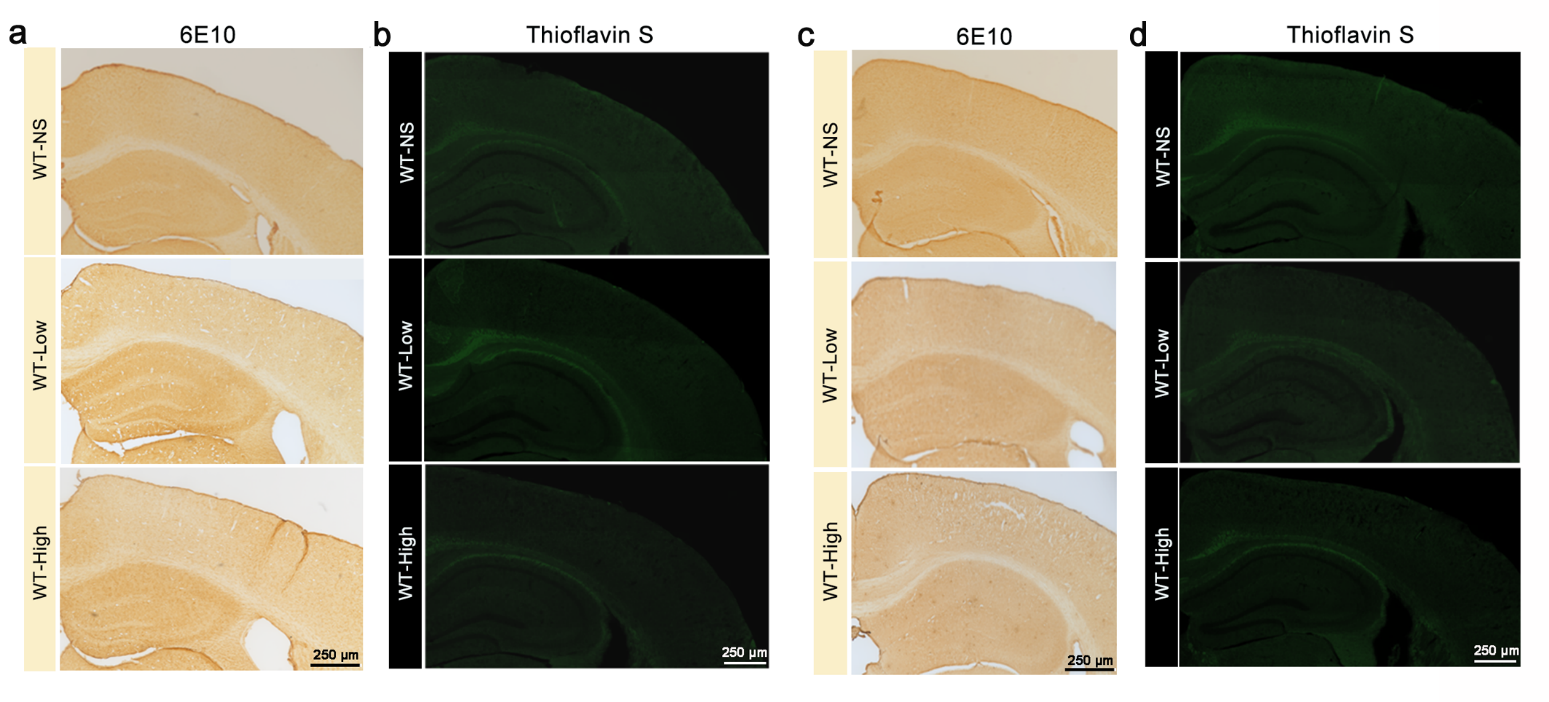
Supplementary Figure S6. No positive staining of 6E10 immunohistochemistry or Thioflavin-S in the brain section of WT mice.**

(a) 6E10 immunohistochemical staining of wild type mice in the prevention experiments.

(b) Thioflavin S staining of wild type mice in the prevention experiments.

(c) 6E10 immunohistochemical staining of wild type mice in the therapeutic experiments.

(d) Thioflavin S staining of wild type mice in the therapeutic experiments.

**
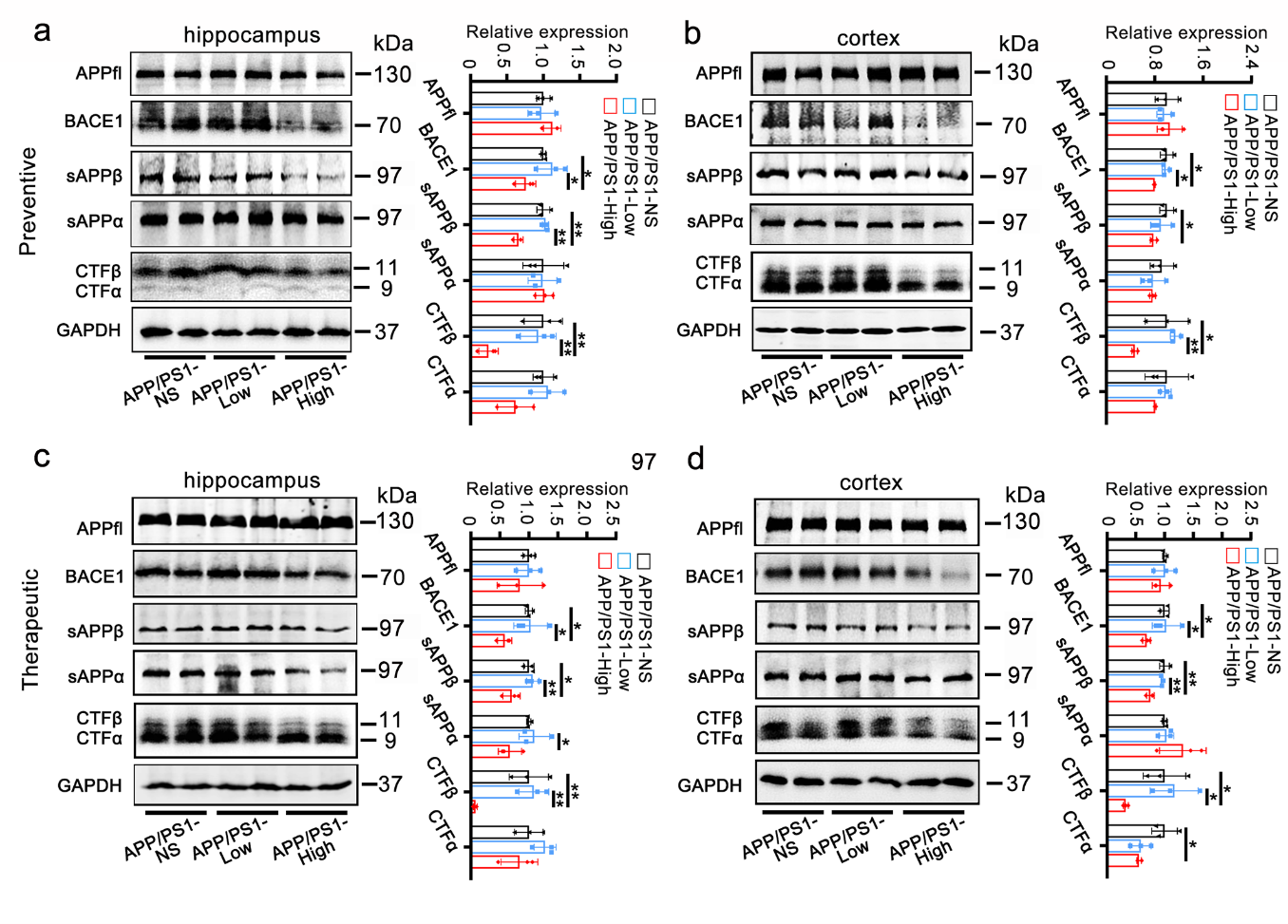
Supplementary Figure S7.** **rhEPO inhibited amyloidogenic processing of APP in APP/PS1 Mice.**

(a-d) Western blotting and quantitative analysis for APP and APP metabolites in the brain homogenates.

(a, b) rhEPO preventive administration reduced Aβ production in the hippocampus (a) and cortex (b).

(c, d) rhEPO therapeutic administration reduced Aβ production in the hippocampus (c) and cortex (d).

Data were presented as mean ±SD. *, *p*<0.05, **, *p*<0.01. N = 3 per group.

**
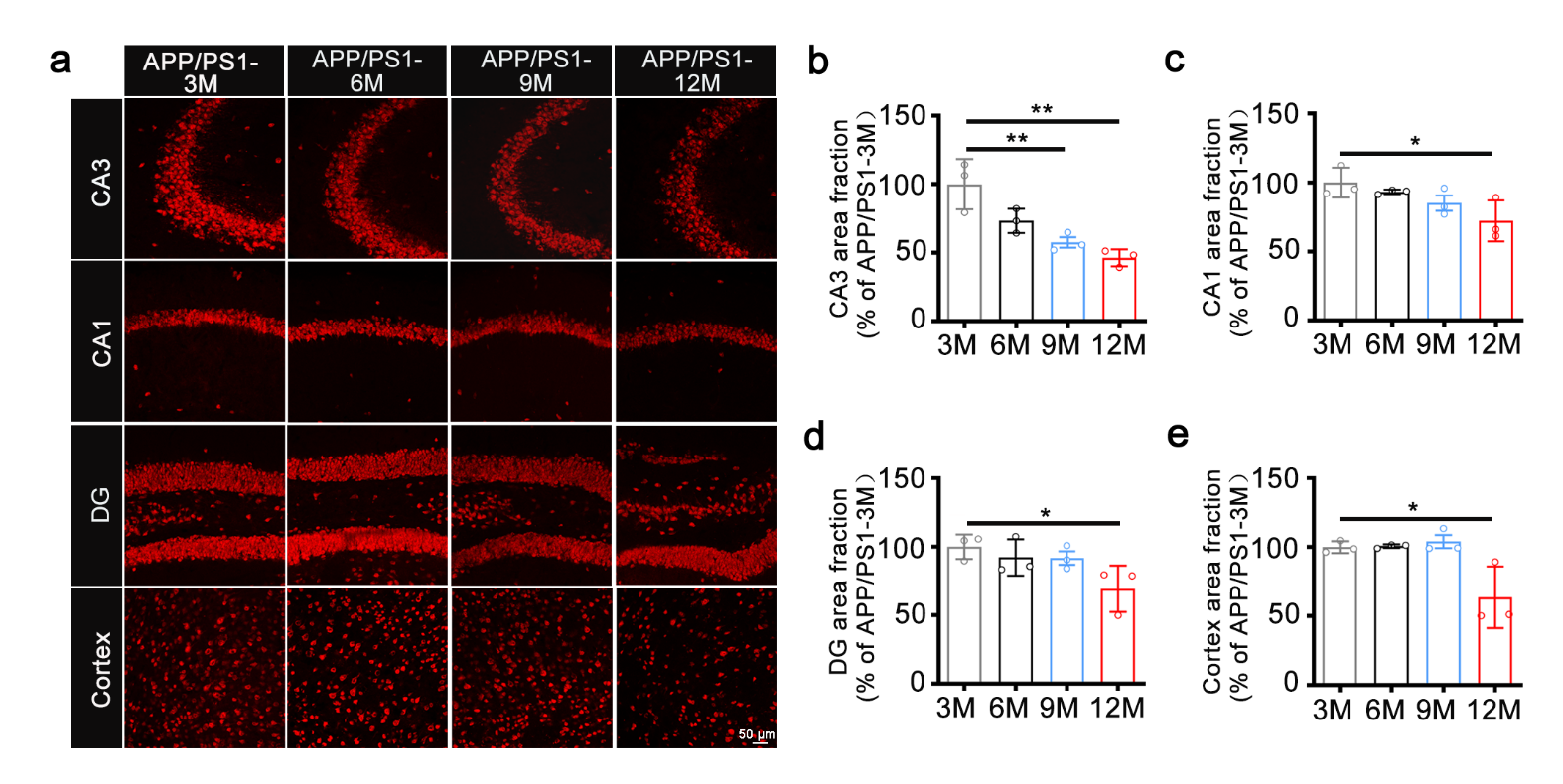
Supplementary Figure S8. NeuN immunofluorescence in age gradient female APP/PS1 mice.**

(a-e) Neuron number in the hippocampal CA1, CA3, DG and cortex of age gradient APP/PS1 mice detected by NeuN immunofluorescence (a) and quantitative analysis (b-e).

Data were presented as mean ±SD. *, *p*<0.05, **, *p*<0.01. N = 3 per group.

**
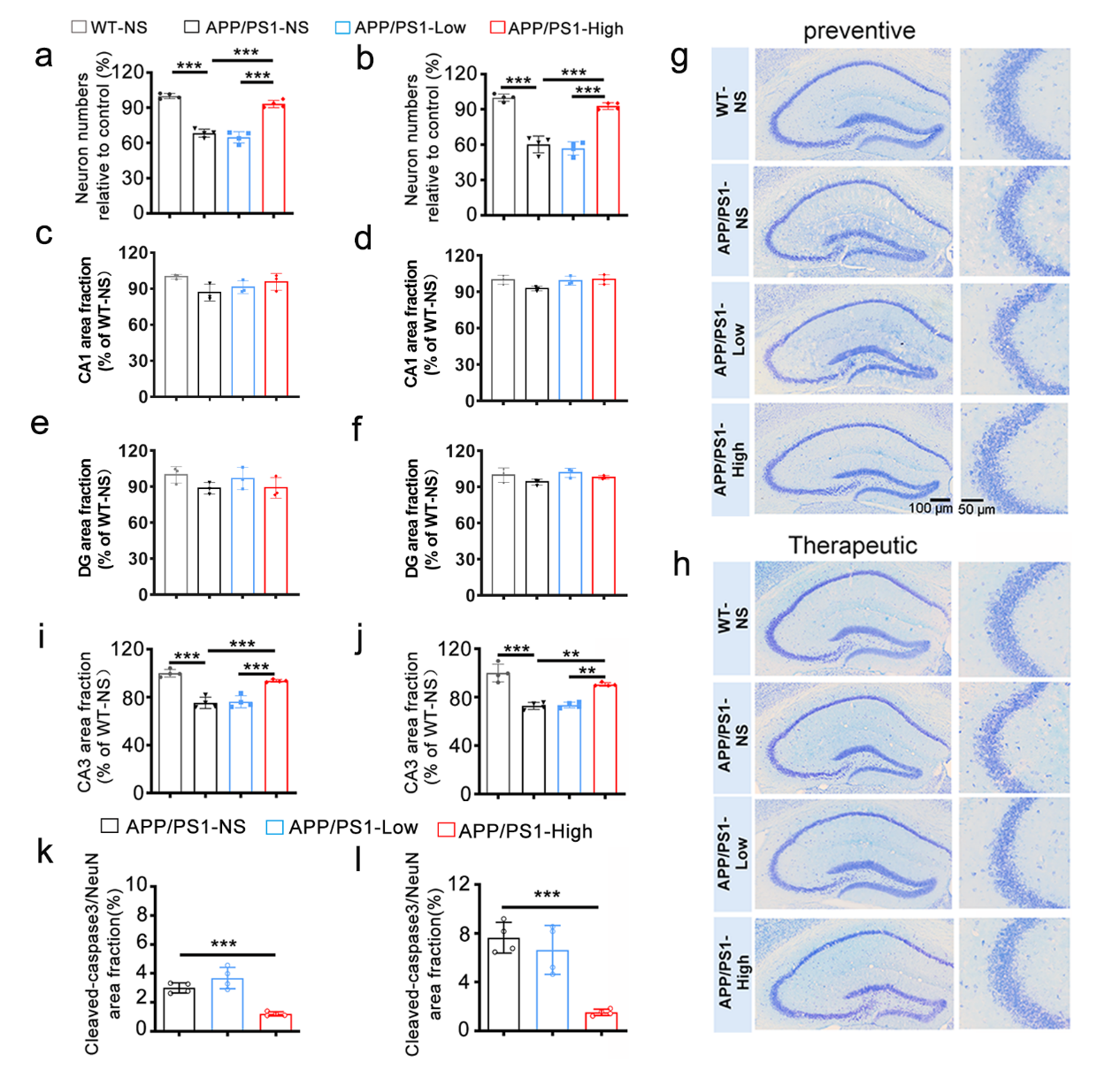
Supplementary Figure S9. rhEPO attenuated neuronal loss in APP/PS1 mice.**

(a-f) Quantitative analysis of NeuN immunostaining in hippocampal CA3, CA1 and DG of the mice in the preventive (a, c, e, image was shown as Fig.1a) and therapeutic experiments (b, d, f, image was shown as Fig.1b).

(g-j) Nissl staining and quantitative analysis in the hippocampus of the mice in the preventive (g, i) and therapeutic experiments (h, j).

(k, l) Quantitative analysis of Neuronal apoptosis detected by immunofluorescence using anti-cleaved-caspase-3 (c-cap3) antibody in hippocampal CA3 in the preventive (k, image was shown as Fig.1c) and therapeutic experiments (l, image was shown as Fig.1d).

Data were presented as mean ±SD. *, *p*<0.05, **, *p*<0.01, ***, *p*<0.001. N = 3 per group.

**
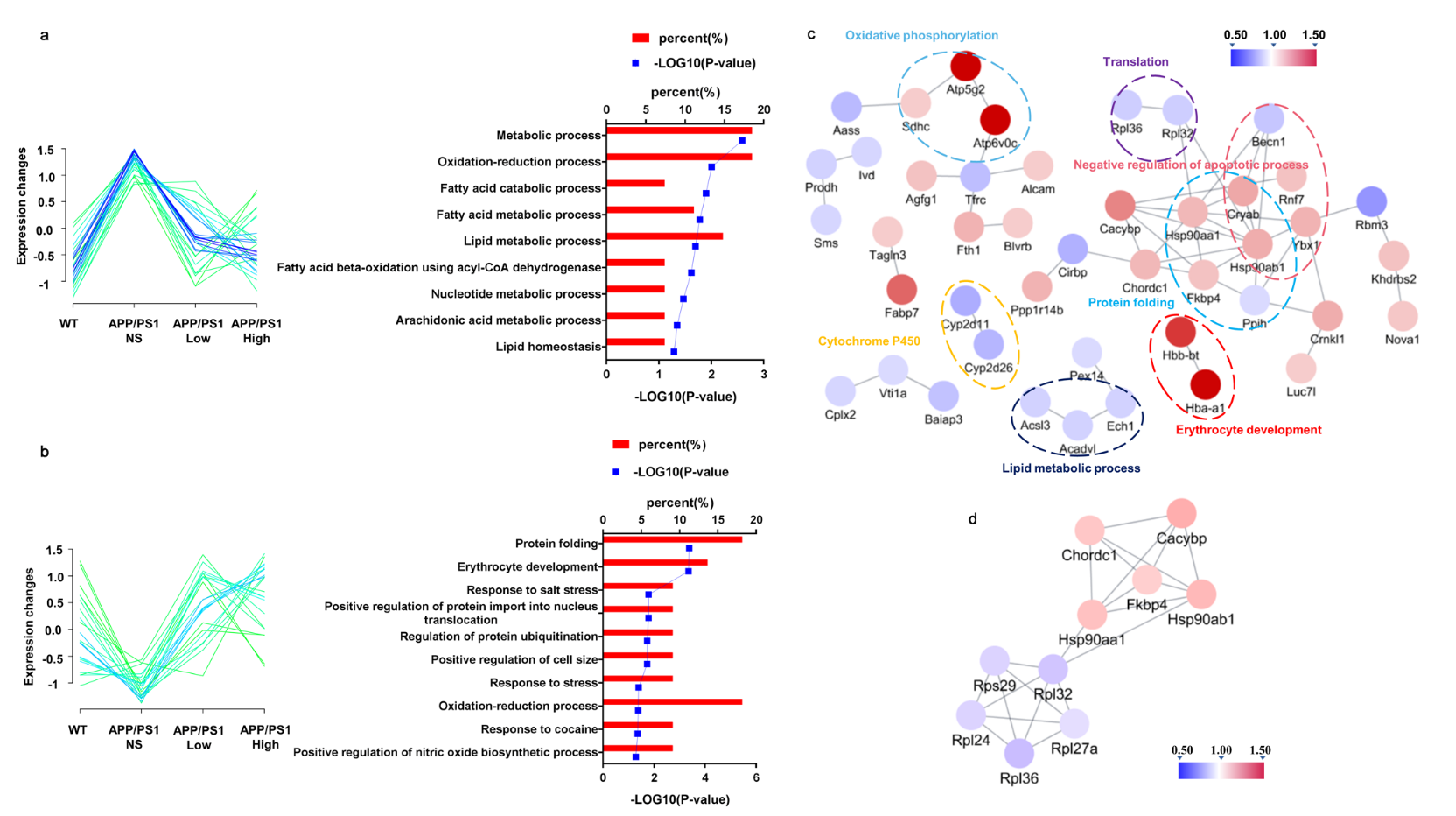
Supplementary Figure S10. rhEPO treatment changed the proteomics profile of hippocampus in APP/PS1 mice.**

(a, b) The reversed expression clusters of DE proteins after rhEPO treatment by Mfuzz analysis and biological process enriched by Gene ontology analysis. The biological processes were enriched *p* value less than 0.05 and top 10 biological processes were showed in graph.

(c) The protein-protein interaction (PPI) map of DE proteins after high dose of rhEPO treatment. The biological functions of the highly correlated proteins were marked with different colors. Red circle represents up-regulation and blue circle represents down-regulation.

(d) MCODE analysis found the closely connected regions in whole PPI map after EPO treatment. Red circle represents up-regulation and blue circle represents down-regulation.

**Supplementary
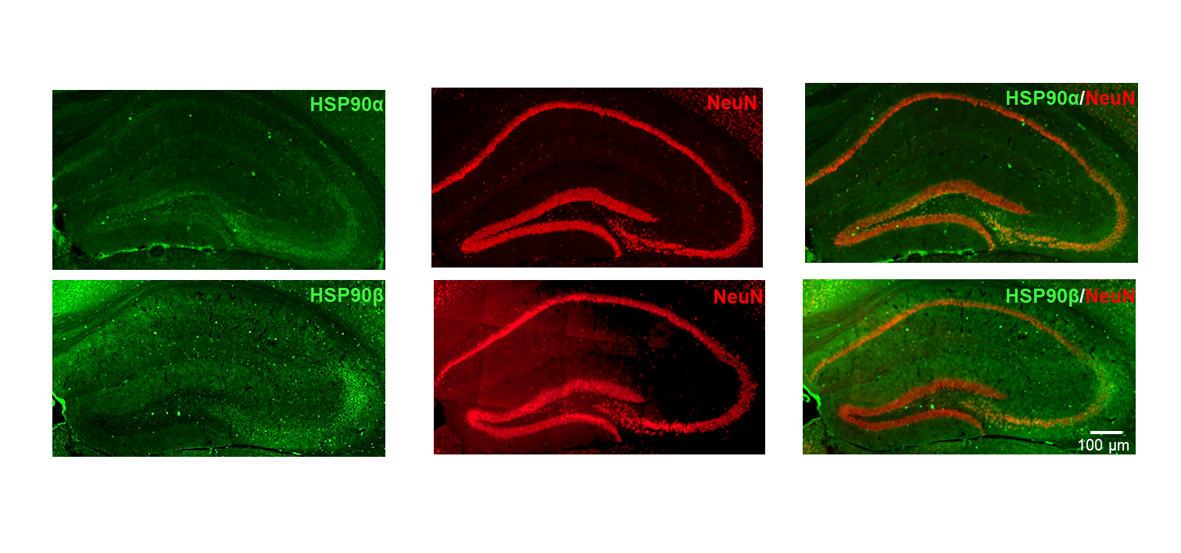
Figure S11. The distribution of HSP90α or HSP90β in the hippocampus.**

The positive immunostaining of HSP90α or HSP90β mainly presented in the hippocampal CA3 region.

**
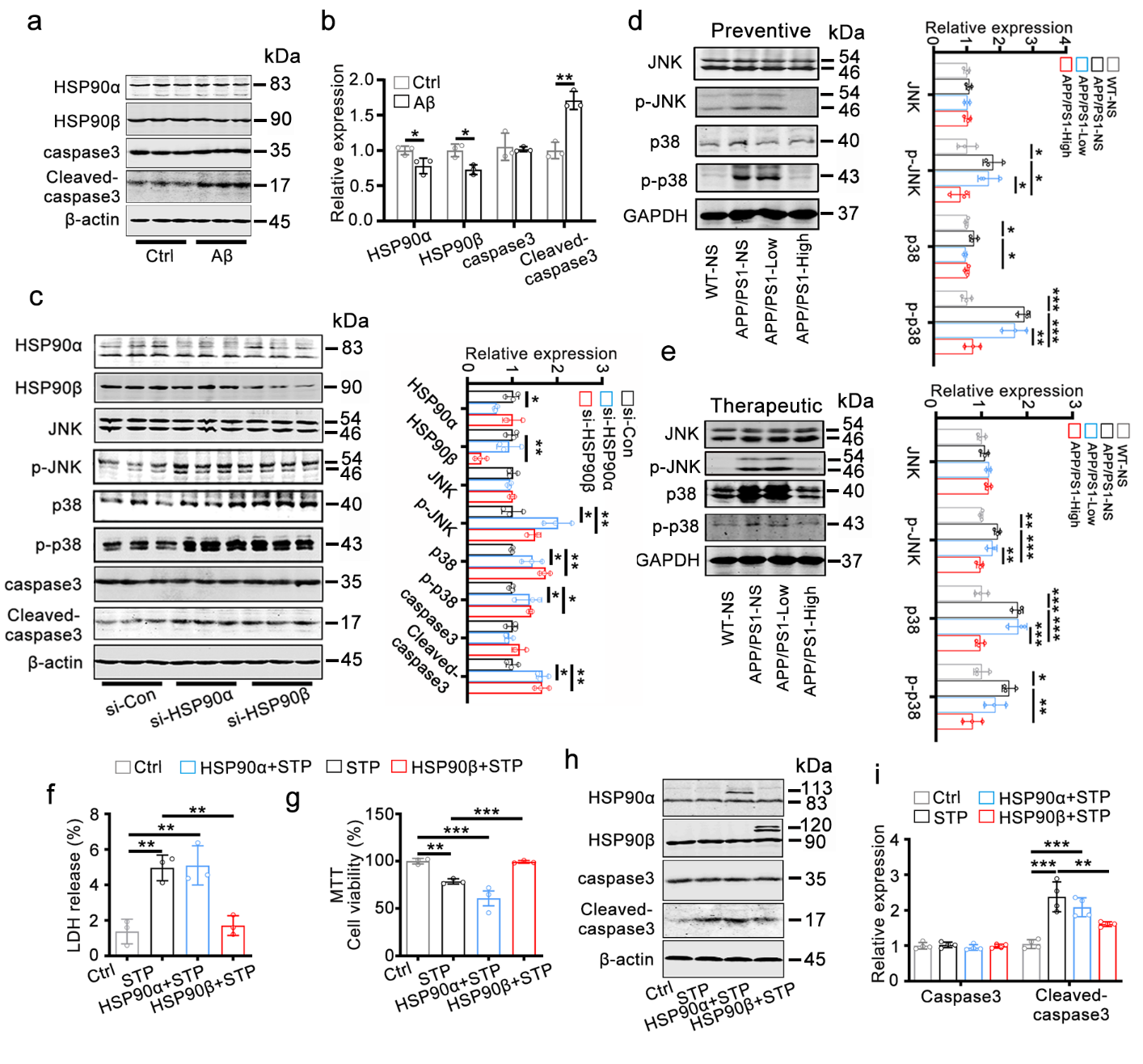
Supplementary Figure S12. Aβ treatment decreased HSP90 level and overexpressing HSP90β attenuated cell apoptosis induced by staurosporine.**

(a, b) Aβ treatment decreased HSP90α and HSP90β levels, and increased cleaved-caspase-3 level in N2a cells.

(c) Knockdown of HSP90α or HSP90β, increased p-JNK, p-p38 or cleaved-caspase-3 expression was detected by Western blotting.

(d, e) Western blotting and quantitative analysis for p-JNK/p-p38 in the hippocampal CA3 in the preventive (d) and therapeutic experiments (e).

(f, g) Overexpression of HSP90β attenuated cell apoptosis induced by staurosporine (STP), which detected by LDH (f) and MTT test (g).

(h, i) Overexpression of HSP90β reduced cleaved-caspase3 level induced by STP.

Data were presented as mean ±SD. *, *p*<0.05, **, *p*<0.01, ***, *p*<0.001. N = 3 per group.


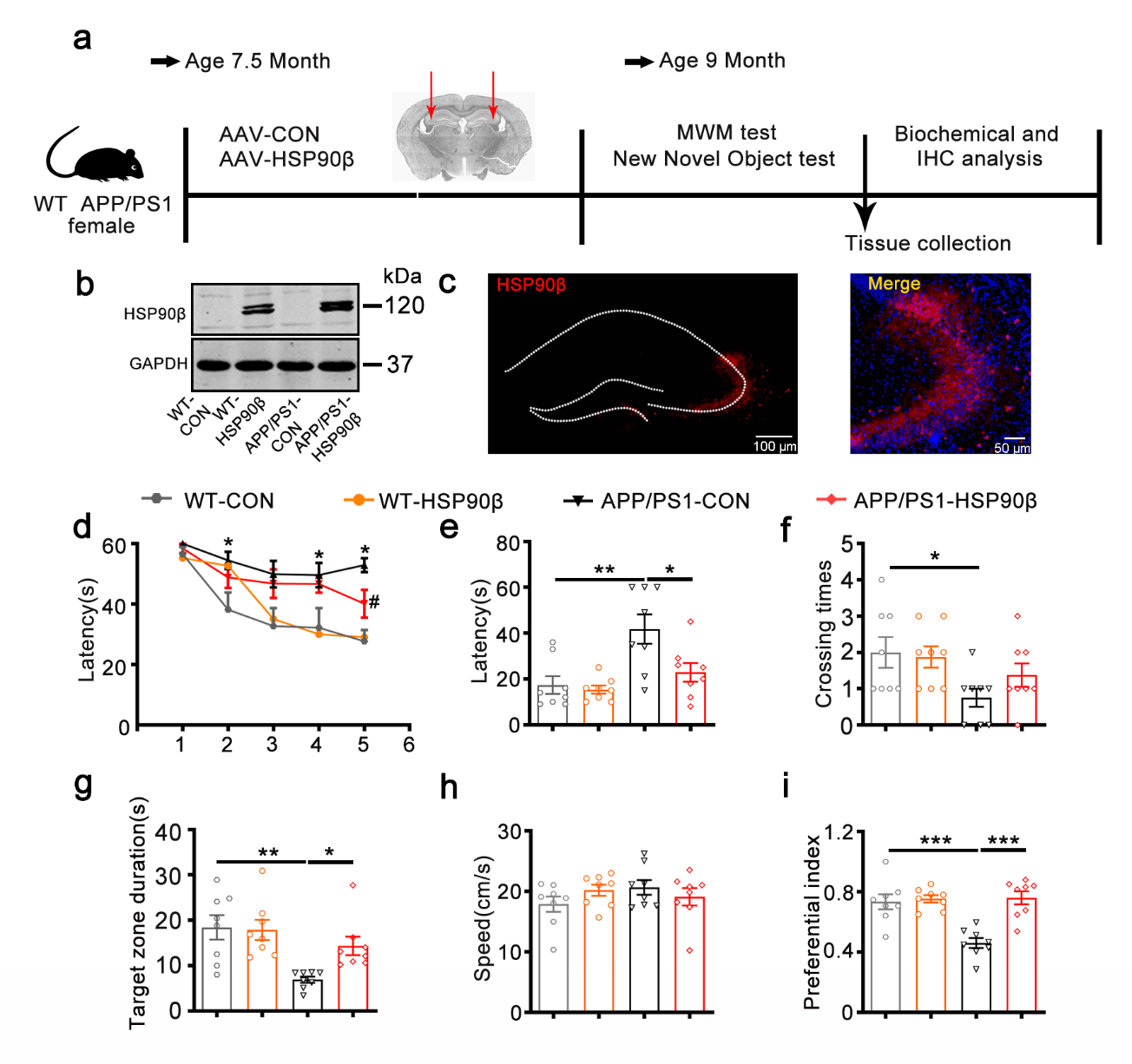
**Supplementary Figure S13. HSP90β overexpression ameliorated memory deficits in APP/PS1 mice.**

AAV-HSP90β-mcherry virus (1.5×10^13^v.g./ml) was stereotaxically injected into the hippocampal CA3 of 7.5-m-old C57 and APP/PS1 mice. The learning and memory ability were detected at 9-month-old.

(a) Experimental schedules for HSP90β overexpression and assessment.

(b, c) Transfected effects of AAV-HSP90β-mcherry virus was confirmed by western blotting and immunofluoresecent staining.

(d) Overexpression of HSP90β ameliorated spatial learning deficits in APP/PS1 mice shown by the decreased escape latency during 5 consecutive days training in Morris water maze (MWM) test.

*, *p*<0.05 *vs* WT-CON; #, *p*<0.05 *vs* APP/PS1-CON. N = 8 per group.

(e-h) Overexpressing HSP90β ameliorated spatial memory deficits in APP/PS1mice shown by the decreased latency to reach the platform site (e), and increased time spent in the target quadrant (g) measured at day 7 by MWM test; HSP90β overexpression had no effect in the crossing time in the platform site (f) and the swimming speed (h). N = 8 per group.

(i) Overexpressing HSP90β ameliorated cognitive impairments of APP/PS1 mice shown by increased time spending in exploring the new novel object measured at 24 h during Novel object recognition test. N = 8 per group.

Data were presented as mean ±SEM. *, *p*<0.05, **, *p*<0.01, ***, *p*<0.001.


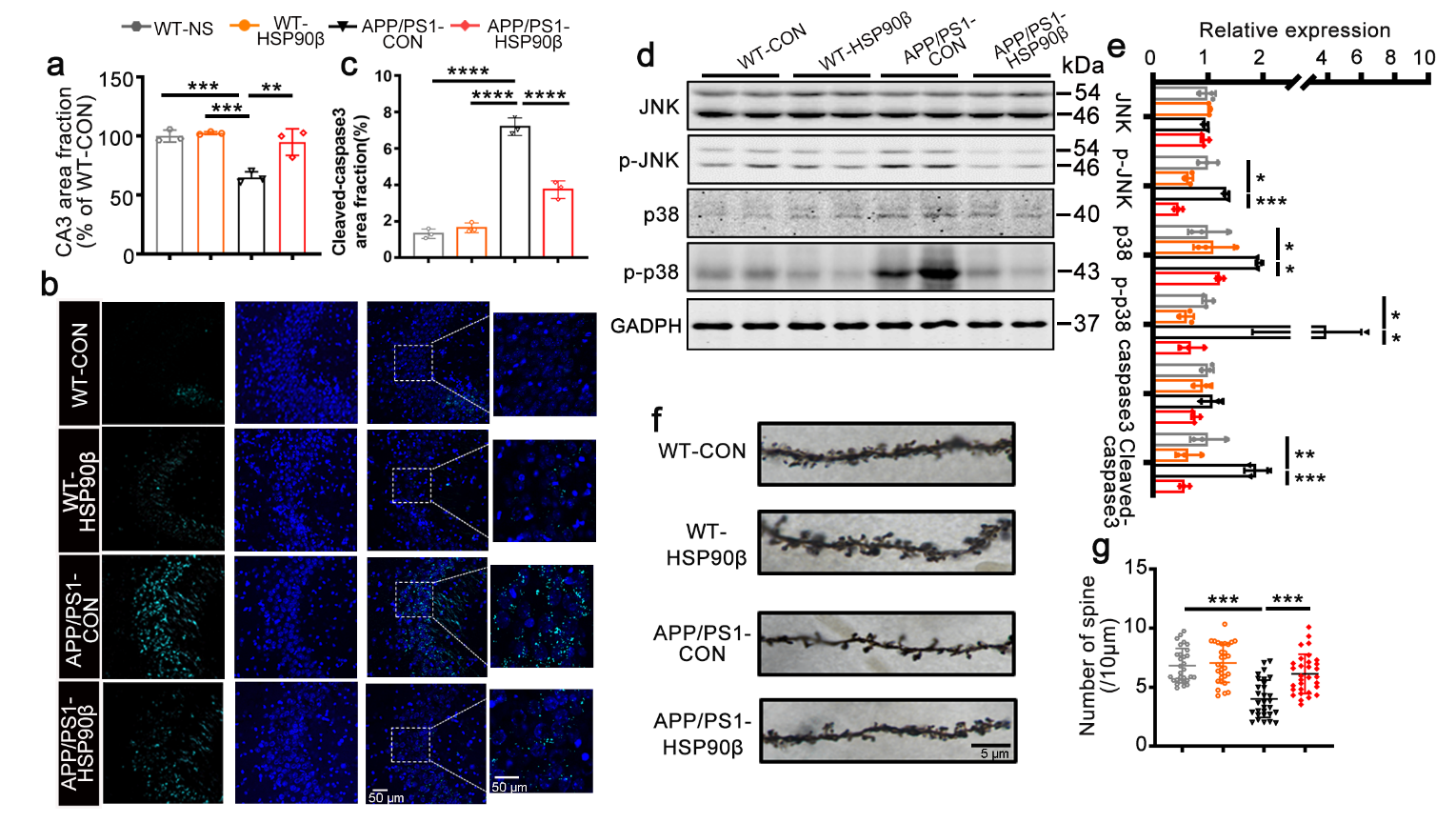
**Supplementary Figure S14. HSP90β overexpression attenuated neuronal loss and synaptic dysfunction in APP/PS1 mice.**

(a) Quantitative analysis of neuronal number detected by Nissl staining in hippocampal CA3 region. Image was shown as Fig. 1s. N = 3 per group.

(b, c) Neuronal apoptosis detected by immunofluorescence with anti-cleaved-caspase-3 antibody in hippocampal CA3 region and quantitative analysis.

(d, e) The levels of JNK, p-JNK, p38, and p-p38 were detected by Western blotting and quantitative analysis. N = 3 per group.

(f, g) Overexpression of HSP90β restored the density of dendritic spine in the hippocampal CA3 neurons of APP/PS1 mice detected by Golgi staining. (n=30 neurons from 3 mice for each group).

Data were presented as mean ±SD. *, *p*<0.05, **, *p*<0.01, ***, *p*<0.001, ****, *p*<0.0001.

**
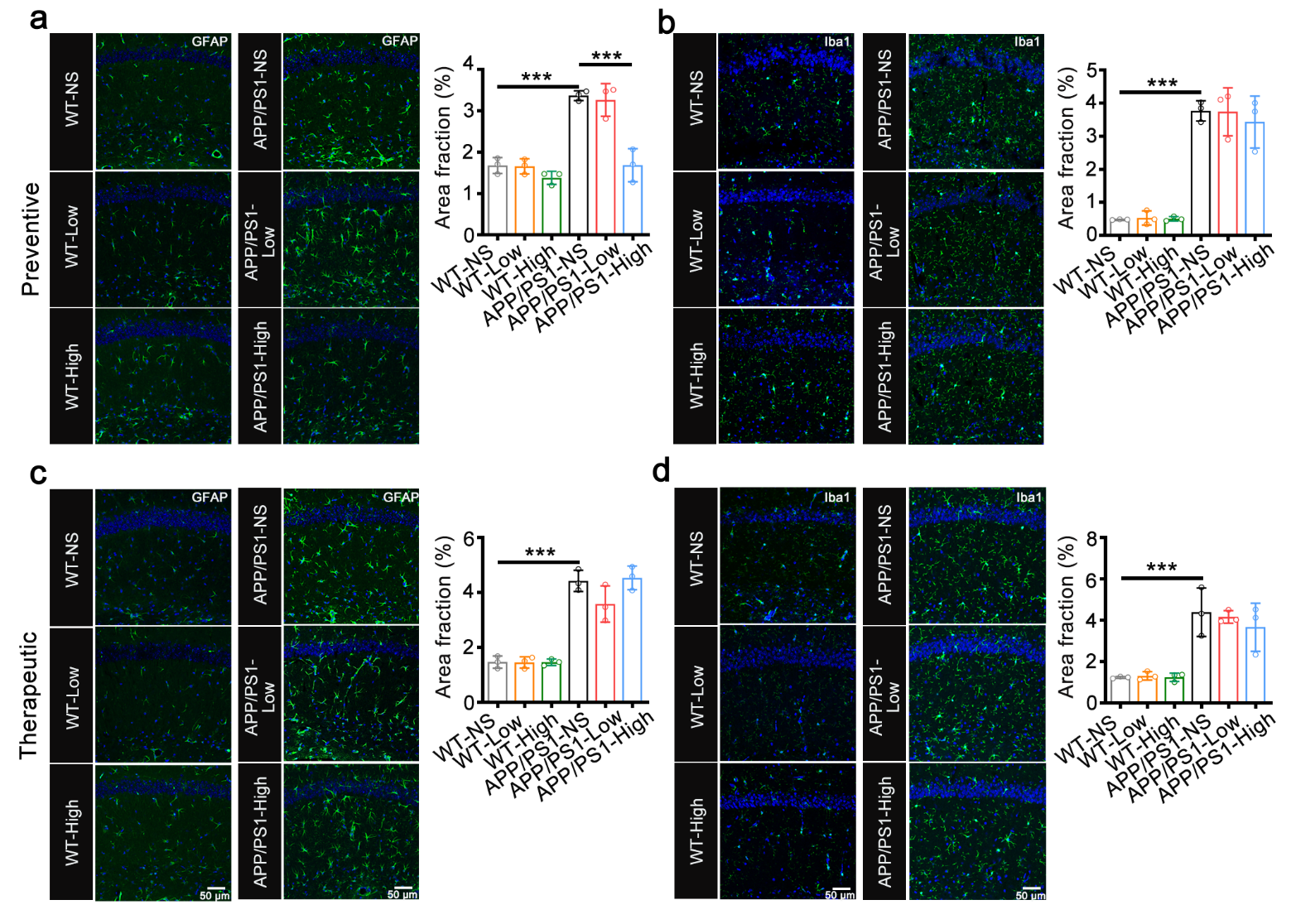
Supplementary Figure S15. Preventive treatment with rhEPO inhibited the activation of astrocytes in the brains of APP/PS1 mice.**

(a, b) Immunostaining and quantification of astrocytosis (anti-GFAP antibody) (a) and microgliosis (anti-IBA1 antibody) (b) in the hippocampus of the preventive experiment.

(c, d) Immunostaining and quantification of astrocytosis (anti-GFAP antibody) (c) and microgliosis (anti-IBA1 antibody) (d) in the hippocampus of the therapeutic experiment.

Data were presented as mean ±SD. ***, *p*<0.001. N = 3 per group.

**
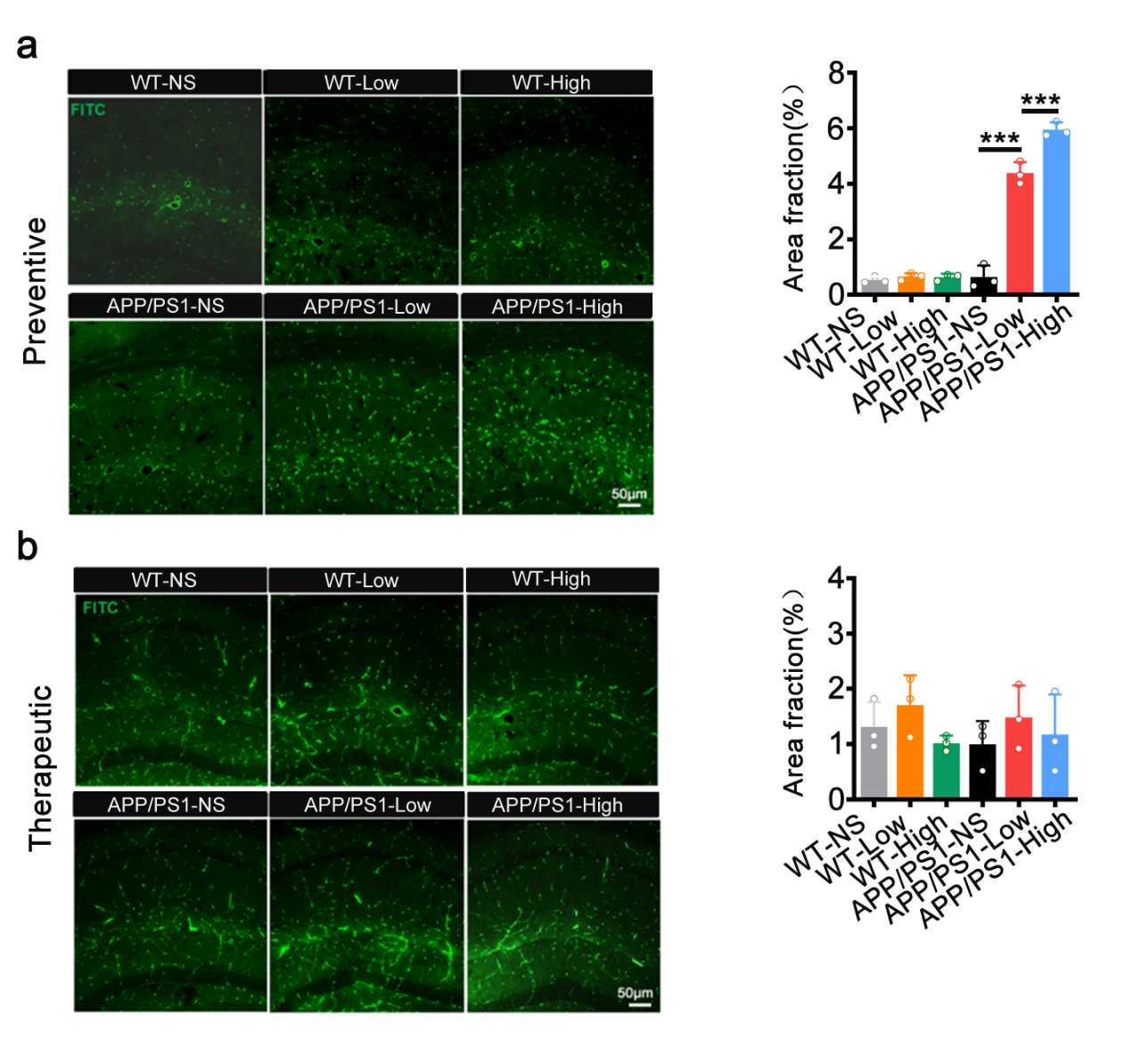
Supplementary Figure S16.** **Preventive treatment with** **rhEPO improved capillary density in the hippocampus of APP/PS1 mice.**

(a) Preventive treatment with rhEPO increased capillary density in the hippocampus of APP/PS1 mice.

(b) Therapeutic treatment rhEPO had no effect in the capillary density in the hippocampus of APP/PS1 mice.

Data were presented as mean ±SD. ***, *p*<0.001. N = 3 per group.

**
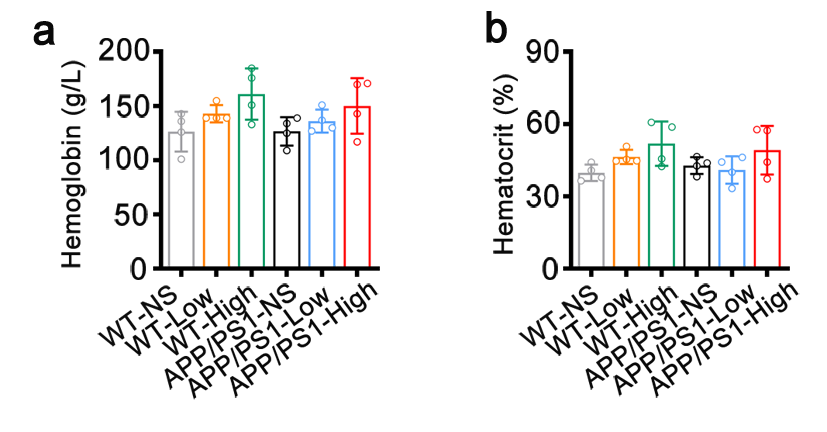
Supplementary Figure S17. Systemically preventive administration with rhEPO had no effect in hematocrit and hemoglobin in peripheral blood.**

rhEPO preventive administration had no effect in hemoglobin (a) and hematocrit (b) in peripheral blood. N = 4 per group.

Data were presented as mean ±SD.

**Supplementary Figure S**
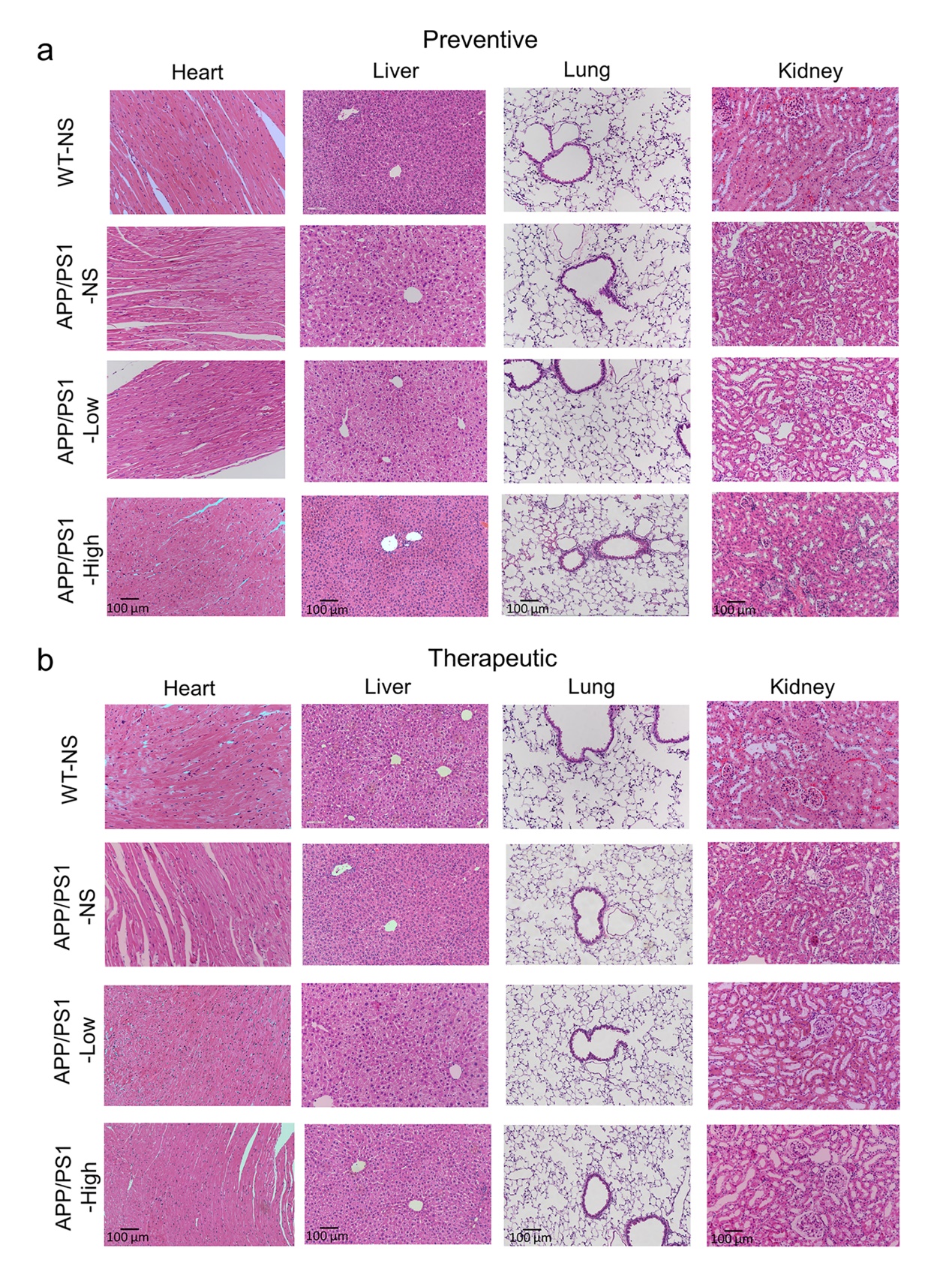
**18.** **There were no side effects after the administration of high-dose rhEPO.**

HE staining for heart, liver, lung, kidney of the mice in the preventive (a) and therapeutic experiments (b).


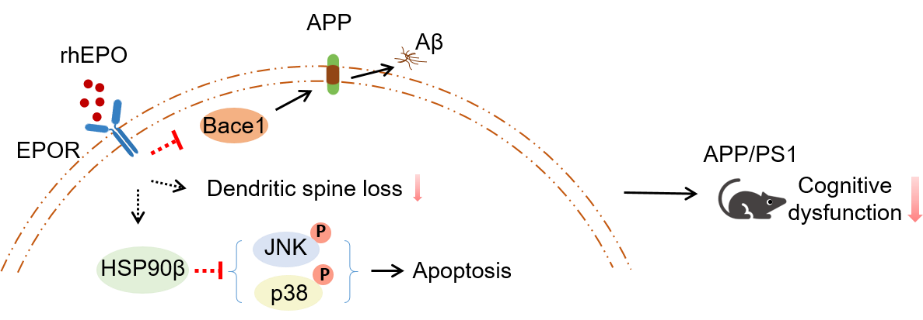
**Supplementary Figure S19. The proposed work model illustrates the mechanism for rhEPO-treated improved the cognition.** rhEPO treatment attenuated JNK/P38 pathway associated apoptosis via upregulation of HSP90β, reduced Aβ load, and reversed dendritic spine loss, together contributed to ameliorate the cognitive impairment of APP/PS1 mice.

**Supplementary Table S1. The antibodies used in the study**

| Antibody | Type | Source |
| --- | --- | --- |
| GAPDH | Mono- | Cell Signaling (Boston, MA) |
| β-Actin | Poly- | Cell Signaling (Boston, MA) |
| HSP90α | Mono- | Abcam (Cambridge, UK) |
| HSP90β | Mono- | Abcam (Cambridge, UK) |
| 6E10 | Mono- | BioLegend (California, USA) |
| APP | Mono- | Sigma-Aldrich (Missouri, USA) |
| APP (recognize APP,  CTF-α, CTF-β) | Poly- | Millpore (Deutschland, Germany) |
| SAPPa | Mono- | IBL (Swit, EUR) |
| SAPPβ | Poly- | IBL (Swit, EUR) |
| BACE1 | Mono- | Cell Signaling (Boston, MA) |
| Caspase-3 | Poly- | Cell Signaling (Boston, MA) |
| Cleaved-Caspase3 | Mono- | Cell Signaling (Boston, MA) |
| GFAP | Mono- | Cell Signaling (Boston, MA) |
| Iba1 | Poly- | Abcam (Cambridge, UK) |
| NeuN | Mono- | Abcam (Cambridge, UK) |
| P38 | Mono- | Cell Signaling (Boston, MA) |
| P-P38 | Mono- | Cell Signaling (Boston, MA) |
| Bcl2 | Poly- | Proteintech (Chicago, IL) |
| BAX | Poly- | Proteintech (Chicago, IL) |
| JNK | Mono- | Sigma(Missouri, USA) |
| P-JNK | Poly- | Abclonal (Boston, MA) |
